# BioSuper: A web tool for the superimposition of biomolecules and assemblies with rotational symmetry

**DOI:** 10.1186/1472-6807-13-32

**Published:** 2013-12-13

**Authors:** Manuel Rueda, Modesto Orozco, Maxim Totrov, Ruben Abagyan

**Affiliations:** 1 Skaggs School of Pharmacy and Pharmaceutical Sciences, University of California, San Diego, 9500 Gilman Drive, La Jolla, CA 92093, USA; 2 Joint IRB-BSC Program on Computational Biology, Institute for Research in Biomedicine, Barcelona Science Park, Josep Samitier 1–5, Barcelona 08028, and Barcelona Supercomputing Center, Jordi Girona 31, Barcelona 08034, Spain; 3 Molsoft L.L.C, 11199 Sorrento Valley Road, S209, San Diego CA 92121, USA

## Abstract

**Background:**

Most of the proteins in the Protein Data Bank (PDB) are oligomeric complexes consisting of two or more subunits that associate by rotational or helical symmetries. Despite the myriad of superimposition tools in the literature, we could not find any able to account for rotational symmetry and display the graphical results in the web browser.

**Results:**

BioSuper is a free web server that superimposes and calculates the root mean square deviation (RMSD) of protein complexes displaying rotational symmetry. To the best of our knowledge, BioSuper is the first tool of its kind that provides immediate interactive visualization of the graphical results in the browser, biomolecule generator capabilities, different levels of atom selection, sequence-dependent and structure-based superimposition types, and is the only web tool that takes into account the equivalence of atoms in side chains displaying symmetry ambiguity. BioSuper uses ICM program functionality as a core for the superimpositions and displays the results as text, HTML tables and 3D interactive molecular objects that can be visualized in the browser or in Android and iOS platforms with a free plugin.

**Conclusions:**

BioSuper is a fast and functional tool that allows for pairwise superimposition of proteins and assemblies displaying rotational symmetry. The web server was created after our own frustration when attempting to superimpose flexible oligomers. We strongly believe that its user-friendly and functional design will be of great interest for structural and computational biologists who need to superimpose oligomeric proteins (or any protein). BioSuper web server is freely available to all users at http://ablab.ucsd.edu/BioSuper.

## Background

The most commonly used way of comparing two biological structures is by calculating their root mean square deviation (RMSD). The RMSD measures the average distance between the atoms in the two structures after optimal rigid body superimposition, yielding a handy single value in distance units. Two identical structures will display a zero RMSD, whereas two distinct ones will display values proportional to their dissimilarity.

Despite its wide use, RMSD calculation still represents a challenge at many levels. For instance, a key step is to establish atomic equivalence in both structures. If the two proteins have identical topology, that is, the atom indexes and names are identical in both coordinate files, then assigning equivalence is trivial. Unfortunately, this is rarely the case (unless we are comparing backbone atoms) and thus some kind of atom mapping is required to establish atom equivalence. In the same context, frequently forgotten issues are resolving positional equivalence of atoms in side chains allowing symmetry ambiguity [1] or accounting for internal symmetry [2-7]. Another key issue is the comparison of proteins with flexible regions or partial overlap, where a direct RMSD calculation provides an unrealistic measure of the similarity. In this regard, several concurrent strategies have been developed under the assumption of assigning greater weight to “rigid” parts of the protein. Their common foundation is to use an iterative search to optimize the superimposition, assigning lower weights to most deviating fragments and thus finding the largest superimposable core [1,8-11]. More important problems arise in the structural comparison of proteins with different sequences. In these cases, a sequence alignment [12] is mandatory before establishing the positional equivalence. In cases where the two proteins have completely different sequences, one must resort to structure-based superimposition methods [13-20].

In macromolecular assemblies, symmetry issues also appear at the protomer level. Most of the proteins in the Protein Data Bank (PDB) are oligomeric complexes consisting of two or more subunits that associate forming rotational or helical symmetries [21-23]. For instance, according to a survey of all *E. coli* proteins (including soluble, membrane-bound, and structural proteins), dimers represented ~ 40% of the species, followed by tetramers (~ 21%), and only ~ 19% were monomers [22]. Among oligomers (up to 12 subunits), homo-oligomers predominate (79%), whereas only 21% form hetero-oligomeric complexes [22]. Protein assemblies having rotational symmetry are classified according to crystallographic point group operations, forming cyclic groups (C*n*), dihedral groups (D*n*) and Icosahedral groups (I*n*). Cyclic groups are the simplest yet most abundant cases among the three in PDB, consisting of a single axis of rotation forming a ring of arranged subunits.

**Figure 1:**
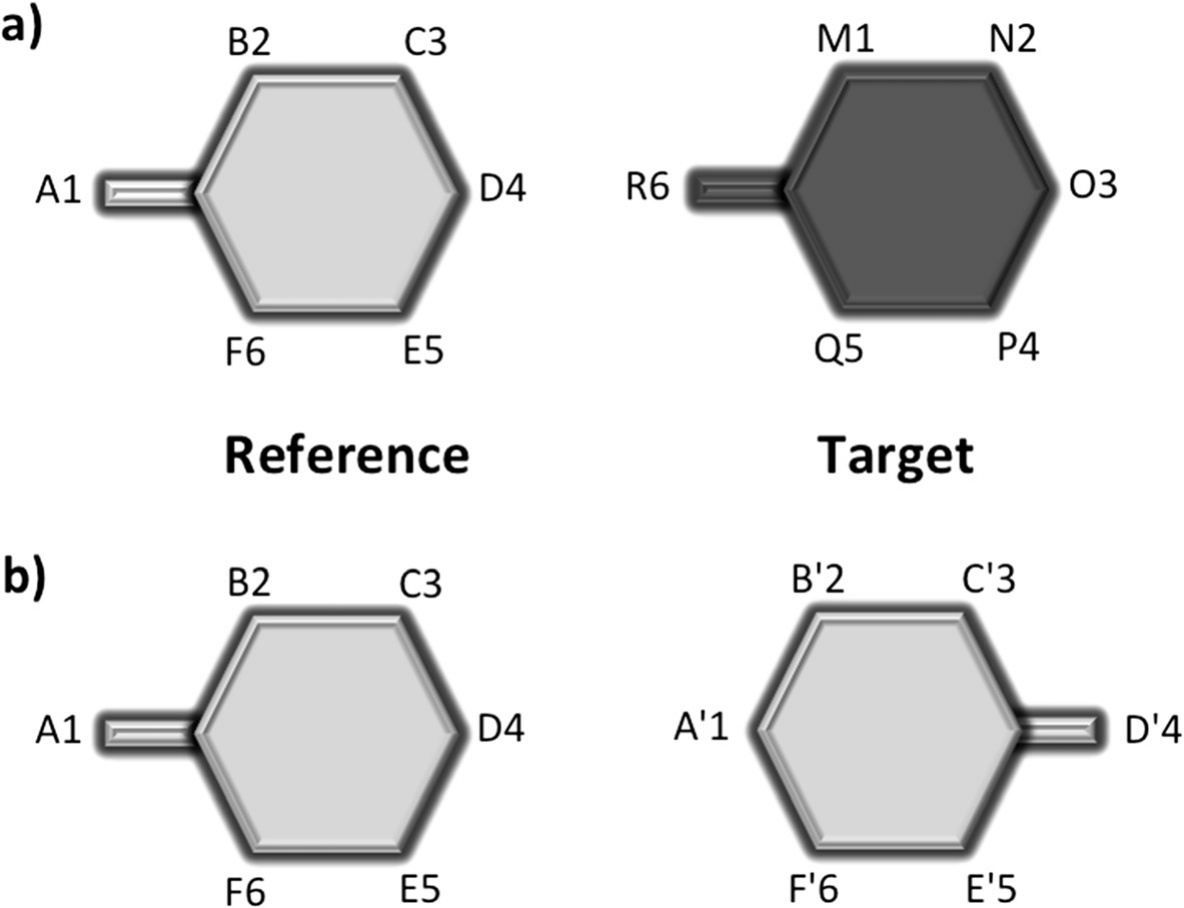
**Schematic representation of the two major issues found when attempting to superimpose protein complexes displaying rotational symmetry: a)** the order of the protomers differs in both complexes; **b)** flexible protomers fluctuate between conformational states.

The superimposition of complexes with perfect rotational symmetry is not an issue because RMSD becomes invariant to rotations. However, symmetry in biomolecules is rarely perfect and often is broken in functionally-relevant conformational changes. For instance, assemblies can adopt *quasi-symmetry*, in which subunits with identical sequence adopt distinct conformations (e.g., flexible proteins fluctuating between states) [24]), or *pseudo-symmetry,* in which different chains form almost symmetrical complexes (e.g., hemoglobin) [22,25,26]. The presence of “imperfect” symmetry (very common in biomolecular systems) generates a hurdle in the superimposition of structures (see Figure 1) by currently available software [14,18,27-31].

Despite the abundance of oligomeric complexes and superimposition tools, after an exhaustive search we could only find one that dealt with the issue of symmetry. The tool is called MultiMeric-Align (MM-align), comes from Zhang’s lab [19] and is an extension of the monomeric alignment program TM-align [9]. MM-align joins individual chains in each complex in every possible order and then finds the optimum structural alignment between them. MM-align is available via source code and web server, and provides basic text information about the final RMSD between complexes, the transformation matrix, as well as a Rasmol script for local visualization of structures. MM-align, although extremely robust, is slow with large proteins and is not very versatile in terms of input and graphical output. For this reason, we decided to extend some of its attributes and develop a new web tool ourselves, taking advantage of all the atom mapping and superimposition features implemented in the ICM modeling platform [32].

Here we present BioSuper, a free web server that allows for fast quaternary structure comparison of homo-oligomeric and hetero-oligomeric proteins displaying *n*-fold rotational symmetry about one axis. BioSuper has built-in *sequence-dependent* and *structure-based* methods for comparison of rigid and flexible proteins, which can be launched simultaneously. The superimposed 3D structures can be interactively manipulated side-by-side in a web browser with the help of a free plugin (or with a free app for the iOS and Android platforms), or downloaded, together with the details describing atomic mappings. This paper describes the implementation of the tool, compares its results with MM-align, and shows a few examples chosen to highlight characteristics that can be of interest for structural and computational biologists.

## Implementation

We created BioSuper as a fast, simple-to-use and platform-independent superimposition tool for structural and computational biologists (or anybody) in need of comparing oligomeric proteins, in particular those forming ring structures. For the client-side operations, we used a responsive design web interface with HTML5 and the *jQuery* JavaScript library, whereas the server-side was implemented using the Perl CGI module. All the core calculations, including sequence alignments [12], atom mapping, superimposition and RMSD calculations are carried out with ICM 3.7 software as described elsewhere [32].

**Figure 2:**
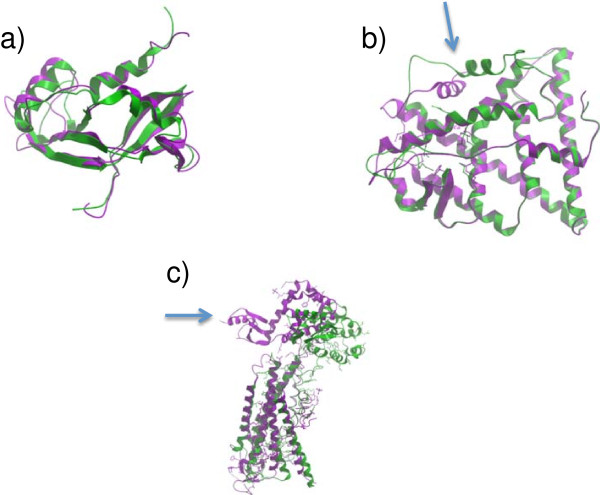
**Examples of the three superimposition types available in the BioSuper web server (**http://ablab.ucsd.edu/BioSuper**): a)***standard* superimposition of the angiogenin protein; PDB IDs: 1agi, chain A (green) and 1gio, chain A from first NMR model (lilac), **b)***weighted* superimposition of the estrogen receptor alpha in different conformations; PDB IDs: 3ert, chain A (green) and 3erd, chain A (lilac), **c)***structural* superimposition of the β_2_ adrenergic receptor and the adenosine A_2A_ receptor; PDB IDs: 2rh1, chain A (green), and 3EML, chain A (lilac).

BioSuper allows for three distinct types of superimpositions, two based on a sequence alignment and one based on a structural alignment. The three superimposition types are part of the ICM distribution and have been exhaustively tested as published elsewhere (see [1]). The simplest, yet the most widely used of the sequence-dependent methods, follows a McLachlan fitting algorithm [33] and is labeled as *standard*. A *standard* superimposition (see example at Figure 2a) works well when the sequences of the proteins to be compared are similar and they do not display flexible domains or regions (e.g., hinges or loops). Using this type of superimposition, equivalence of symmetric side chain atoms in Phe, Arg, Asp, Glu, Leu, Phe, Tyr, and Val residues is taken into account when the user selects the mode *heavy-atoms*. For proteins having similar sequences but displaying flexible regions or partial overlap, a Gaussian-weighted iterative approach (labeled as *weighted*) [11] can be used to find the best superimposable core in both structures. The idea is that non-flexible atoms will have a greater weighting than those that move (see Figure 2b). In cases where the sequences of both proteins differ, a *structure-based* method (labeled as *structure*) is the most convenient (see Figure 2c)*.* This approach finds the residue-to-residue correspondence based on the Cα coordinates. The structural alignment algorithm is based on the ZEGA (zero-end-gap-alignment) dynamic programming procedure [13,34].

**Figure 3:**
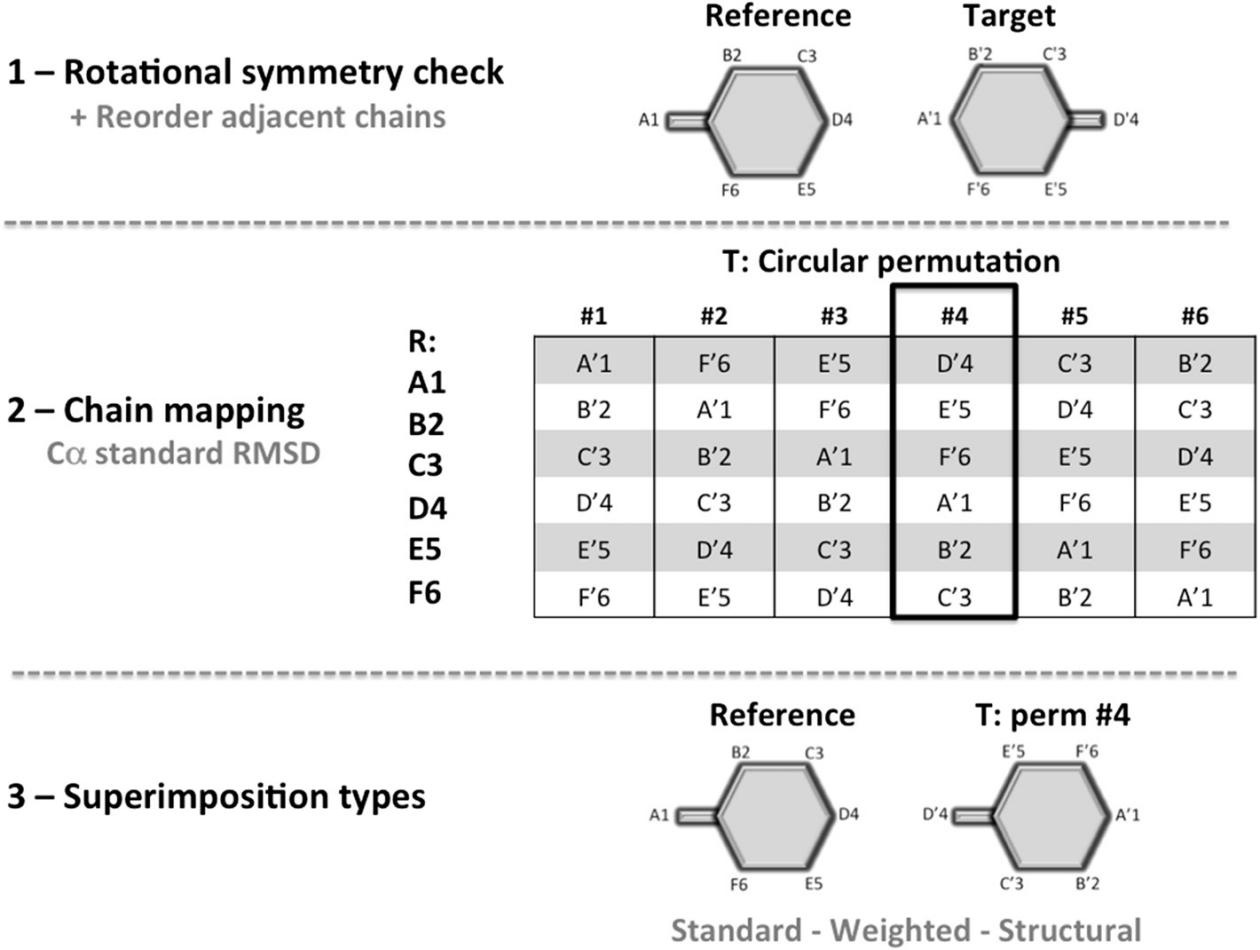
**Overview of BioSuper web server approach.** Whenever the server finds that the protein target has multiple chains, it triggers a series of events that end up in finding the optimum superimposition of protein assemblies displaying rotational symmetry.

Prior to any of the three mentioned superimposition types, if the *target* consists of multiple chains, the server performs the following operations:

1. The server performs *N* x *N* (*N* being the number of chains in the target) sequence alignments to look for chain replicas. In the event that a given chain from the target displays a sequence identity ≥ 95% (threshold extracted from http://www.pdb.org[35]) with any other chain from the same structure, the server assumes that rotational symmetry exists (see Figure 3). Note that the user can modify this threshold. By default, the server reorders the chains in the pdb files so that adjacent ones become consecutive (for the reference and the target). The idea is to consider as adjacent the chain that is closer in space. For instance, taking the first chain (A’) as a reference, the server computes the distances between its center of mass and the center of mass of the remaining chains, and adds as a consecutive (A’ + 1) the chain having the minimum distance. The procedure is continued after all chains have been processed without allowing repetitions. This fast operation is able to correctly reorder the vast majority of assemblies checked, however, it can fail with hetero-oligomers (e.g., the biological unit of the PDB ID 1hho) or with complex dihedral symmetries. For this reason, it can be turned *on* or *off* by the user*.* As a rule of thumb, we always recommend to manually verify chain position in the structures when working with biological units.

2. A search is performed to establish the chain mapping that minimizes the *standard* Cα RMSD between the reference and the target. The chains of the target are mapped to those in the reference by performing a clockwise shifting, as follows (see Figure 3): if we assume that the reference has 3 chains labeled as: “che-mis-try” (where each letter can be understood as an amino acid sequence or as a 3D feature) and the target has 3 chains labeled as “cal-che-mi”, two clockwise permutations of the target chains lead to “mi-cal-che” and “che-mi-cal”. In this case, “che-mi-cal” permutation will lead to the optimum chain mapping.

3. The 3D coordinates of the optimal permutation will be used for any of the 3 superimposition types available.

It is worth noting that we do not perform any rotation about an angle during the search, but an optimal circular permutation. In contrast to MM-align, BioSuper does not find the best mapping of each individual chain from the target on the reference. This decision was deliberate to avoid the steric clashes that appear in the interfaces after individual superimposition of chains. The method was originally intended for assemblies displaying cyclic groups but it also works with many oligomers displaying dihedral groups (a dihedral group contains an axis of rotational symmetry and a perpendicular axis of two-fold symmetry; see examples in the next section). The server was not built to superimpose complexes displaying helical symmetry and thus it is not suitable for viral capsids, etc.

## Results and discussion

### Input and graphic visualization

The structures can be retrieved by their PDB code (6) or uploaded as PDB files. The user has the option of selecting individual protein chains and the level of atom selection (Cα, backbone, or heavy-atom). As an added feature, BioSuper has the option of constructing the biological units (biological assemblies) from the asymmetric ones. This operation can be performed only if the pdb contains appropriated information in the REMARK 350 tag (more information in the help section at the web site). The user also can activate or deactivate the chain reordering feature, and can change the sequence identity threshold for considering two chains as equal.

**Figure 4:**
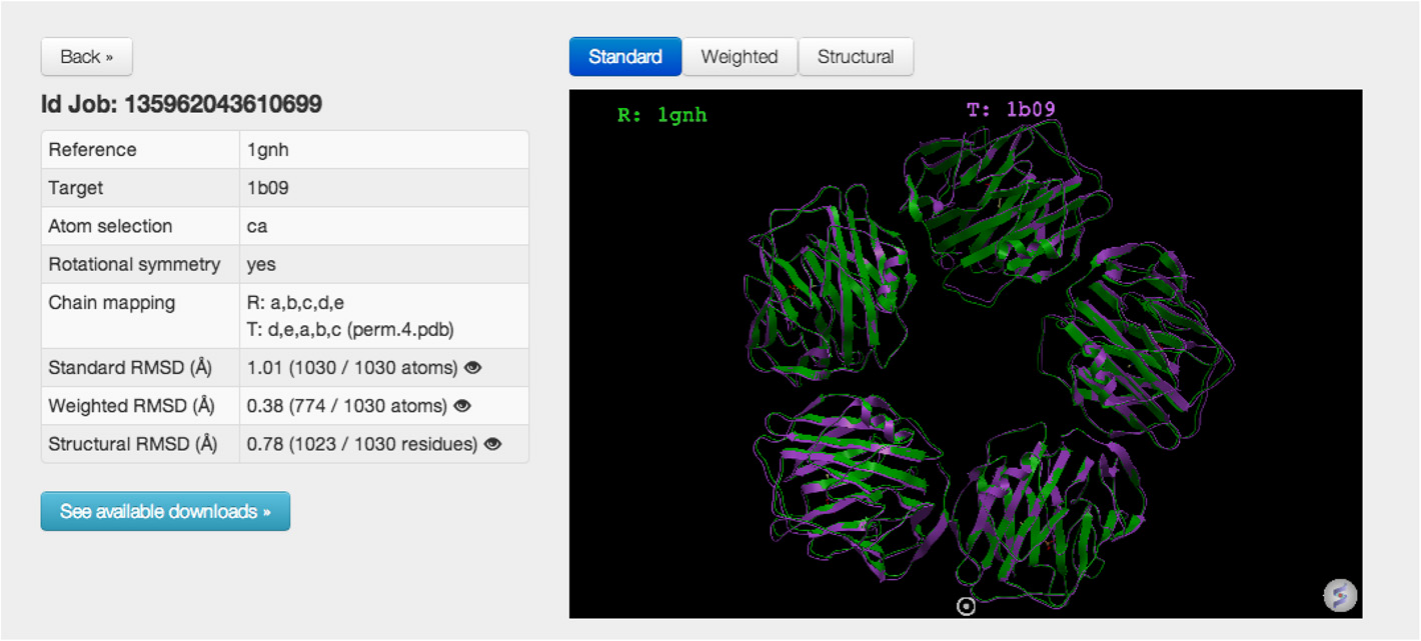
**Example of BioSuper’s main page of results.** The data are shown via HTML tables plus interactive 3D graphics via the ActiveICM free plugin [36]. Note that the user can easily switch superimpositions by clicking a button. The page also displays information on reference and target structures, atom selection, presence of rotational symmetry, chain and atom mappings, the RMSD values, as well as buttons for file download.

The results are presented immediately after hitting the submit button, with an average execution time of ~ 1 second (per superimposition type) for average-sized proteins, and of 2–30 seconds for large biological units (5×-10× faster than MM-align). The results are primarily web-based, consisting of HTML tables containing all numeric results and atom mapping, plus embedded 3D interactive molecular objects (see Figure 4). To visualize the 3D objects inside the browser the user needs to install the free activeICM/active X plugin (7). Alternatively, the 3D objects can be downloaded to be manipulated with the free ICM browser (for desktops or laptops computers) or with the free iMolview Lite (Android and iOS devices). The server also provides the option of downloading superimposed PDB structures, as well as all possible chain mappings as text files (viewable by most modeling programs) for further manipulation or analysis. All the information contained in the HTML tables can also be downloaded as a comma separated value (.csv) text file. The server includes a simple mechanism to try out sample data, a help page with extended documentation on the technical details, and several pre-computed examples showing scenarios where it can be applied (e.g., homo-oligomers, hetero-oligomers, quasi-symmetry and pseudo-symmetry).

### Benchmark

We conducted a benchmark to compare the performance of BioSuper and MM-align. Prior to the comparison, we want to emphasize that our goal with BioSuper was not to create “another” structural superimposition tool, or to compete with any existing one, but rather to fill a gap existing for comparison of quaternary structures of symmetric assemblies.

**Figure 5:**
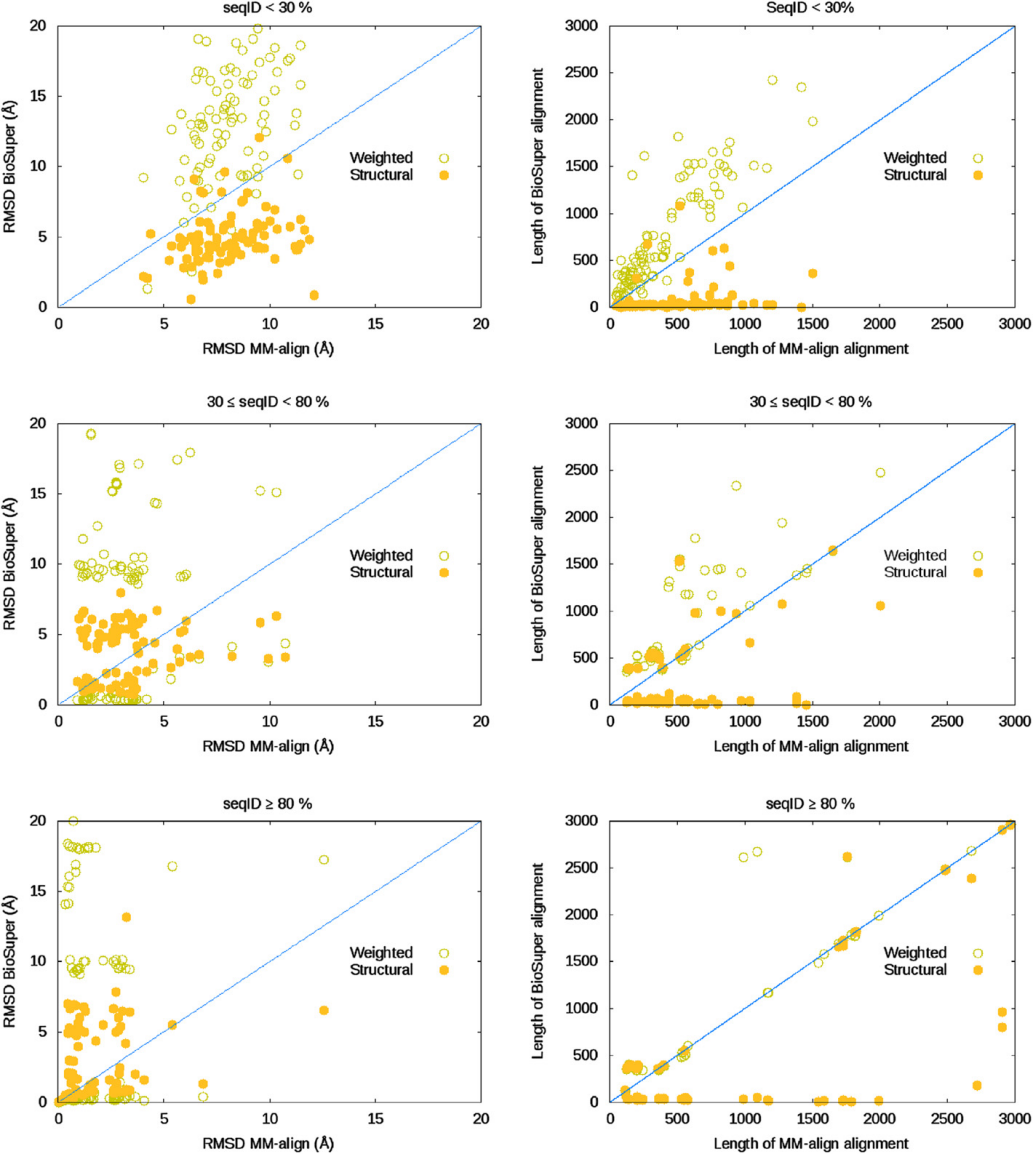
**Comparison between MM-align and BioSuper on C6 structures downloaded from the Protein Data Bank.** Every dot in the scatter plots represents one of the 300 randomly selected pairwise superimpositions between the 205 structures. The plots on the left show the BioSuper RMSD values (according to the *weighted* and *structural* superimpositions) vs. MM-align values. The plots on the right correspond to the number of aligned residues on every superimposition. The plots on the top correspond to C6 assemblies having a sequence identity < 30%, the plots on the middle correspond to pairs with 30% ≤ sequence identity < 80% and the plots in the bottom correspond to pairs with sequence identity ≥ 80%.

We performed a search in the PDB and downloaded all the PDB IDs having global C6 symmetry (248 PDB IDs as September 2013) to be later be used in pairwise superimpositions. We selected C6 complexes due to the relative low number of structures available, yet we think the conclusions can be extrapolated to any other symmetry type. From these 240 structures, we narrowed the selection to those assemblies that yielded 6 chains in the biological unit (205). Our objective was to classify pairs by sequence similarity, so, for each protein we joined the 6 chains into a single one and performed 20910 possible pairwise sequence alignments. 20147 (96%) had a sequence identity < 30%, 229 (0.01%) had 30% ≤ sequence id. < 80, and 534 (0.03%) had a sequence identity ≥ 80%. From each of these 3 subsets we selected 100 random pairs and performed calculations with a standalone version of MM-align and BioSuper web server (via Perl LWP script). Every subset had diverse representation of protein sizes, ranging from 125 to 4000 residues. In Figure 5, we compare RMSD values obtained from BioSuper with respect to RMSD values from MM-align, together with the number of residues aligned. Ideally, we would like an approach that aligns the maximum number of residues while keeping the RMSD distances to minimum values. For BioSuper we reported the weighted RMSD (sequence-dependent) and the structure-based RMSD.

As expected, for protein pairs having a sequence identity < 30%, a superimposition based on sequence alignments did not provide optimum results, and thus the weighted RMSD values were higher that those coming from the structural alignment of MM-align (see Figure 5). Interestingly, the number of aligned residues when using a sequence-based superimposition was consistently higher than that based on structure from MM-align. Part of this effect is due to the superior accuracy of the “chain-to-chain” alignments in BioSuper, with respect to the “joined-chains” ones in MM-align. BioSuper’s structural superimposition provided lower RMSD values than MM-align, but only because ICM aligned fewer residues than MM-align. Complexes having sequence identity between 30% and 80% provided overall better RMSDs values in both programs. This time BioSuper’s weighted superimposition performed remarkably well in many cases, yet still aligning more residues than MM-align. As before, the BioSuper structural RMSD values were lower that those from MM-align, due to the lower number of aligned residues. BioSuper provided the best results in terms of aligned residues when the sequence of both complexes was ≥ 80%. The weighted superimposition was able to align the same or more residues than MM-align, thus providing more realistic RMSD values than purely structural ones. The fact that structure is more conserved than sequence became apparent, and weighted RMSD values were still higher than the structural alignment from MM-align. In case of structure-based superimpositions, MM-align provided larger alignments yet its RMSD values were comparable to those from BioSuper.

In summary, we can conclude that BioSuper is able to correctly map the chain correspondence between two protein assemblies displaying rotational symmetry, and its “chain-to-chain” sequence alignment has greater accuracy than the “joined-chains” one. This is particularly important when comparing assemblies with similar sequences that are fluctuating between conformational states. BioSuper calculation times were on average 5×-10× faster than those from MM-align. On the other hand, in cases where the sequence of both complexes differs, the structural alignment from MM-align is able to align more residues (according to its text-based output) yet keeping decent RMSD values.

### Examples with proteins displaying rotational symmetry

#### C-reactive protein

**Figure 6:**
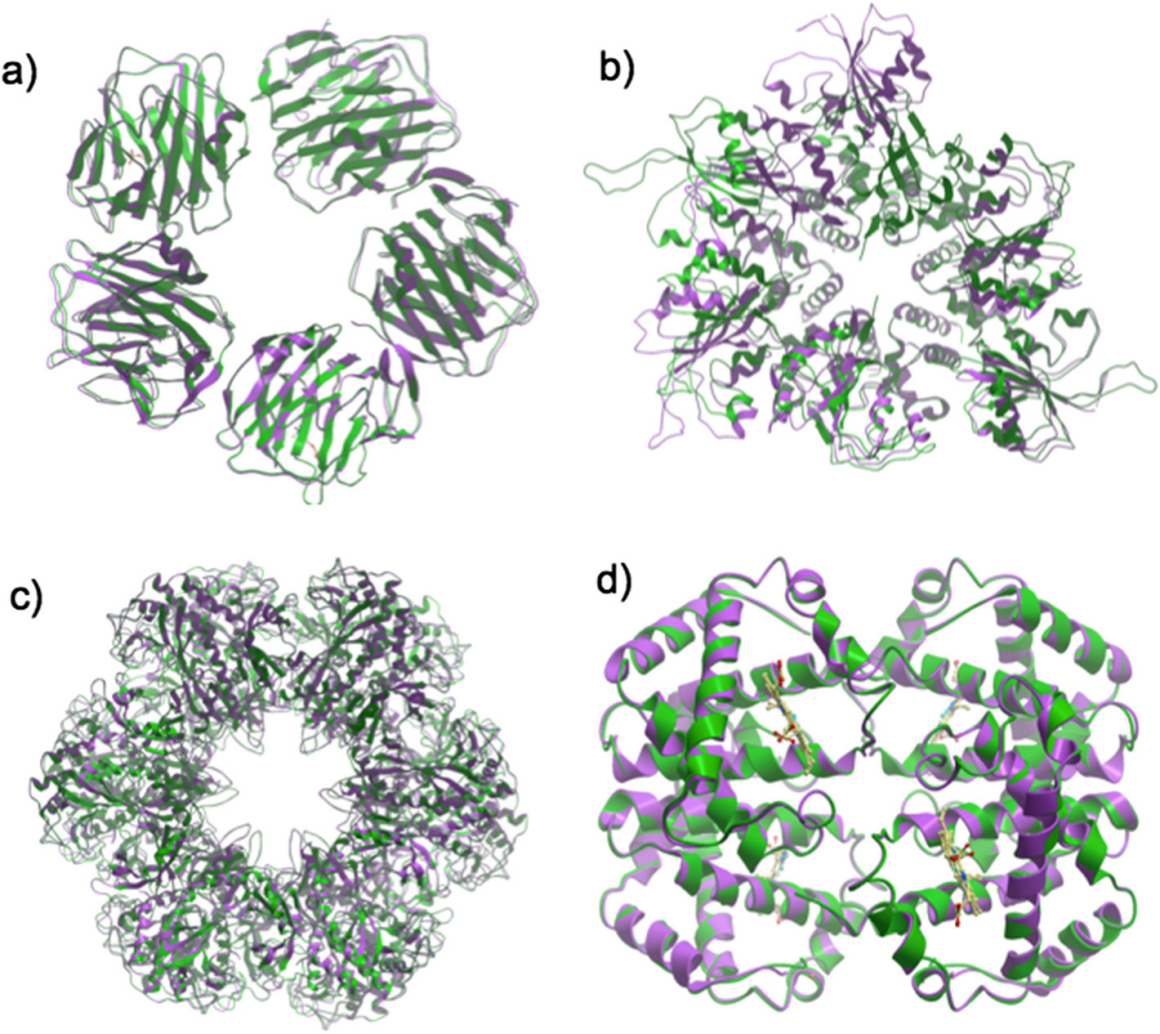
**Examples of superimposition of proteins displaying rotational symmetry. a)***Standard* superimposition of the C-reactive protein (CRP) PDB IDs: 1gnh (green) and 1b09 (lilac). The quaternary structure consists of five identical chains (homo-oligomer) and is classified as a cyclic group C5. The optimal permutation of chains was: R: a,b,c,d,e; T: d,e,a,b,c and led to a standard RMSD of 1.01 Å (for the 1030 Cα atoms), **b)***Weighted* superimposition of the protein RepB, PDB IDs 3dkx (green) and 3dky (lilac). The quaternary structure consists of 6 identical chains (homo-oligomer), being classified as cyclic groups C2 and C3, respectively. Because of the high flexibility of the protein, the *weighted* RMSD provided a better overall superimposition, and led to a weighted Cα RMSD of 0.92 Å (536/1159 atoms), **c)***Standard* superimposition of the Glutamine Synthethase Class I (GSI) from *Salmonella typhymurium*, PDB IDs 1fpy (green) and 1f1h (lilac). The quaternary structure consists of 12 identical chains (homo-oligomer) arranged as a ring and classified as dihedral group D6. In this case, the optimal permutation of chains was that in the PDB files, that led to a *standard* RMSD value of 0.71 Å (5304/5616 Cα atoms), **d)***Standard* superimposition of human deoxyhemoglobin, PDB IDs 1fdh (green) and 2hhb (lilac). The quaternary structure is a tetrameric hetero-oligomer (α_2_β_2_) and is classified as a cyclic group C2. The results revealed that the optimal permutation of chains was: R: a,g,b,h; T: a,b,c,d that led to an *standard* RMSD value of 0.42 Å (574 Cα atoms).

The C-reactive protein (CRP) is a protein found in the blood that is involved in the activation complement system via the C1Q complex. The quaternary structure consists of five identical chains (homo-oligomer) and is classified as a cyclic group C5. For such a protein, we chose PDB IDs that had identical sequence but displayed a conformational change (quasi-symmetry) (reference: 1gnh and target: 1b09). In this case, the optimal permutation of chains was: R: a,b,c,d,e; T: d,e,a,b,c that led to an standard RMSD of 1.01 Å (for the 1030Cα atoms; see Figure 6a). The original chain mapping led to a Cα RMSD value of 1.47 Å. For this protein, a *standard* superimposition yielded optimum results.

#### RepB

RepB is the initiator of DNA replication of streptococcal RCR plasmid pMV158. The structure of native full-length RepB reveals a hexameric ring molecule, where each protomer has two domains organized as a toroid [24]. The catalytic domains appear to be highly mobile and RepB hexamer (RepB_6_) has been crystallized as trigonal and tetragonal forms (PDB IDs: reference: 3dkx and target: 3dky), displaying C2 and C3 cyclic groups respectively (quasi-symmetry). In this case, after creation of the biological units, the optimal permutation of chains was that present in the original x-ray structures. Such chain mapping led to a *standard* RMSD value of 9.92 Å (1159/1161 Cα atoms). Because of the high flexibility of the loop portion, the *weighted* RMSD led to a Cα RMSD value of 0.92 Å (536/1159 atoms below 2 Å) and provided a better overall superimposition (see Figure 6b).

#### Glutamine Synthetase Class I

Glutamine Synthethase Class I (GSI) from *Salmonella typhymurium* is an enzyme that plays an essential role in the metabolism of nitrogen by catalyzing the condensation of glutamate and ammonia to form glutamine. The quaternary structure consists of 12 identical subunits (homo-oligomer) arranged as a ring classified as dihedral group D6. For this example, we chose two PDB IDs that displayed quasi-symmetry (reference: 1fpy and target: 1f1h). In this case, the optimal permutation of chains was that present in the original PDB files, that led to a *standard* RMSD value of 0.71 Å (5304/5616 Cα atoms; see Figure 6c). For this protein, a *standard* superimposition type yielded optimum values.

#### Hemoglobin

Adult human hemoglobin is a α_2_β_2_ tetrameric (hetero-oligomer) hemeprotein present in erythrocytes, responsible for binding oxygen in the lung and transporting the bound oxygen throughout the body. The quaternary structure of the protein displays rotational symmetry and is classified as a cyclic group C2. For this application, we chose two PDB IDs that displayed pseudo-symmetry, the human deoxyhemoglobin (reference: 1fdh and target: 2hhb). The results revealed that the optimal permutation of chains was again that present in the original x-ray files that led to a *standard* RMSD value of 0.42 Å (574 Cα atoms; see Figure 6d). For this protein a *standard* superimposition led to optimum results.

## Conclusions

We have developed a web tool named BioSuper that allows for fast and easy superimposition of proteins and assemblies displaying rotational symmetry about one axis. To the best of our knowledge, Biosuper is the first web tool of its kind that provides instantaneous visualization of the 3D graphical results in the browser, biomolecule generator capabilities, as well as sequence-dependent and structure-based superimpositions, and the only web tool that takes into account atomic equivalence of atoms in side chains displaying symmetry ambiguity. The web server was created after our own frustration when attempting to superimpose snapshots coming from molecular dynamics simulations of flexible oligomers, and we believe that it can also be extremely useful to crystallographers and computational biologists.

### Availability and requirements

● Project name: BioSuper

● Project home page: e.g. http://ablab.ucsd.edu/BioSuper

● Operating system(s): Platform independent

● Programming languages: Perl, HTML5, JavaScript.

● Other requirements (free 3D visualizators): ActiveICM (browser plugin) or iMolview Lite (iOS and Android apps)

● License: GNU GPL (Perl scripts)

● Any restrictions to use by non-academics: None.

## Competing interests

The authors declare that they have no competing interest.

## Authors’ contributions

MR contributed to the design, implementation and testing of the web server, and wrote the manuscript. MO was involved in revising the manuscript. MT and RA wrote the ICM program subroutines that are used by the web server, plus RA revised the manuscript. All authors read and approved the final manuscript.
